# The Effect of Azithromycin on Ivermectin Pharmacokinetics—A Population Pharmacokinetic Model Analysis

**DOI:** 10.1371/journal.pntd.0000236

**Published:** 2008-05-14

**Authors:** Ahmed El-Tahtawy, Paul Glue, Emma N. Andrews, Jack Mardekian, Guy W. Amsden, Charles A. Knirsch

**Affiliations:** Global Clinical Research, Pfizer, New York, New York, United States of America; Task Force for Child Survival and Development, United States of America

## Abstract

**Background:**

A recent drug interaction study reported that when azithromycin was administered with the combination of ivermectin and albendazole, there were modest increases in ivermectin pharmacokinetic parameters. Data from this study were reanalyzed to further explore this observation. A compartmental model was developed and 1,000 interaction studies were simulated to explore extreme high ivermectin values that might occur.

**Methods and Findings:**

A two-compartment pharmacokinetic model with first-order elimination and absorption was developed. The chosen final model had 7 fixed-effect parameters and 8 random-effect parameters. Because some of the modeling parameters and their variances were not distributed normally, a second mixture model was developed to further explore these data. The mixture model had two additional fixed parameters and identified two populations, A (55% of subjects), where there was no change in bioavailability, and B (45% of subjects), where ivermectin bioavailability was increased 37%. Simulations of the data using both models were similar, and showed that the highest ivermectin concentrations fell in the range of 115–201 ng/mL.

**Conclusions:**

This is the first pharmacokinetic model of ivermectin. It demonstrates the utility of two modeling approaches to explore drug interactions, especially where there may be population heterogeneity. The mechanism for the interaction was identified (an increase in bioavailability in one subpopulation). Simulations show that the maximum ivermectin exposures that might be observed during co-administration with azithromycin are below those previously shown to be safe and well tolerated. These analyses support further study of co-administration of azithromycin with the widely used agents ivermectin and albendazole, under field conditions in disease control programs.

## Author Summary

This paper describes the use of a modeling and simulation approach to explore a reported pharmacokinetic interaction between two drugs (ivermectin and azithromycin), which along with albendazole, are being developed for combination use in neglected tropical diseases. This approach is complementary to more traditional pharmacokinetic and safety studies that need to be conducted to support combined use of different health interventions. A mathematical model of ivermectin pharmacokinetics was created and used to simulate multiple trials, and the probability of certain outcomes (very high peak blood ivermectin levels when given in combination) was determined. All simulated peak blood levels were within ranges known to be safe and well tolerated. Additional field studies are needed to confirm these findings.

## Introduction

The operational efficiency of disease elimination programs in developing countries could be improved by integrating delivery of several interventions at local (village and district) levels [1]–[3]. In areas endemic for co-infection with filarial nematodes and Chlamydia trachomatis, one such integrated disease elimination strategy would be based on mass administration of a three-drug combination: ivermectin for onchocerciasis, albendazole for lymphatic filariasis and azithromycin for trachoma. Regular administration of this combination would also be predicted to reduce other infectious agents including soil transmitted nematodes and bacterial sexually transmitted diseases [4].

A recent pharmacokinetic study evaluated co-administration of azithromycin, ivermectin and albendazole [5], and showed that mean ivermectin pharmacokinetic parameters, area under the concentration-time curve (AUC) and maximum concentration (Cmax), were increased by 31% and 27%, respectively relative to a baseline period. The variability in this interaction was large, with two individuals having 3-fold increases in ivermectin AUC. Increased ivermectin exposures could potentially have safety implications, as high dose ivermectin animal studies and observations of human overdose have reported signs and symptoms of central nervous system (CNS) toxicity including emesis, mydriasis and ataxia [6]. However a recent safety study demonstrated no significant toxicity in the CNS or other body systems, with ivermectin doses up to 10 times the highest labeled dose of 200 µg/kg [7],[8].

The purpose of this analysis was to model the ivermectin pharmacokinetic data from the recently reported interaction study [5], to further characterize the interaction, and explore the sources of variabilities between subjects and across treatments. The model was also used to simulate the outcomes of 1000 trials, to ensure that peak ivermectin exposures seen during co-administration did not exceed those observed in the high dose safety and pharmacokinetic study [7].

## Methods

### Study Design and Data Assembly

Data from a historical Phase I study with intensive sampling in healthy subjects was used to develop a population pharmacokinetic model for ivermectin [5]. All subjects provided written informed consent according to local requirements before entering the study, and the protocol and Informed Consent Form were approved by the local Institutional Review Board. This was a three-arm crossover study, where subjects were administered single-dose regimens of the following treatments in random order: (i) azithromycin 500 mg; (ii) ivermectin 200 µg/kg of total body weight rounded to the nearest 3 mg plus albendazole 400 mg; and, (iii) all 3 drugs administered concurrently. All doses were administered with 240 mL of water and a standardized breakfast. Prior to dosing and breakfast, subjects fasted overnight and then abstained from any further food for 4 hours after study drug administration. Study arms were separated by washout periods of 3 weeks. Full details of the study are provided in [5].

**Figure 1:**
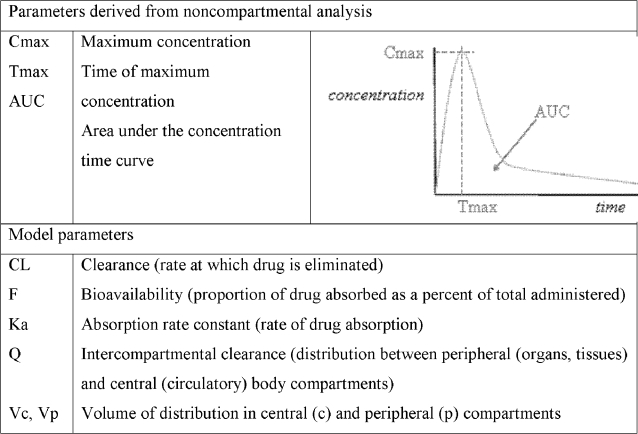
Key pharmacokinetic parameters.

Blood samples were collected predose and at 0.5, 1, 1.5, 2, 3, 4, 6, 8, 10, 12, 24, 36, 48, 72, 96, 120, 144, and 168 hours after drug administration during each of the study phases. Samples were collected into heparinized Vacutainers. Blood samples were centrifuged at 3000 rpm for 15 minutes and the plasma samples were collected in plain plastic tubes without anticoagulant and then stored at −80°C. Samples were shipped frozen overnight on dry ice to BAS Analytics (West Lafayette, IN) for sample analyses. Ivermectin is detected in the body as two metabolites (22,23-dihydroavermectin-B1a (H2B1a) and 22,23-dihydroavermectin-B1b (H2B1b), and these were assayed using a validated high performance liquid chromatography system with liquid chromatography/mass spectrographic detection. The assays were linear over the ranges of 2.5–1000.0 ng/mL and 2.5–20.0 ng/mL, respectively. The precision values for both assays were <10%. In terms of accuracy, while the bias was not exceeded (±15%) for H2B1b for either the high or low quality control (QC) samples, they were for H2B1a during long-term stability testing (−21.8% at the low QC and −17.3% for the high QC) (see [5]). Plasma concentration-time data were analyzed using standard noncompartmental analytical software (WinNonlin 4.1; Pharsight Corporation, Mountain View, CA), and key parameters are shown in Figure 1. The data analysis presented here is for ivermectin data from the ivermectin plus albendazole arm (Baseline Phase), and from the ivermectin, albendazole plus azithromycin arm (Interaction Phase).

Eighteen healthy Caucasian volunteers were enrolled in and completed this study (9 males and 9 females, mean [±SD] age, 39.4±10.5 years, weight 78.2±12.4 kg, ivermectin dose 15.5±2.6 mg).

### Pharmacokinetic Modeling

All the data from both arms of the cross-over study were fitted simultaneously. The data set contained pooled pharmacokinetic, demographic/covariate, and dosing information. Data were analyzed using nonlinear mixed-effects modeling with the NONMEM software system, Version V, Level 1.1 (GloboMax LLC, Ellicott City, MD) with the PREDPP model library and NMTRAN subroutines. Computer resources included personal computers with Intel Pentium 4 processors, Windows XP Professional operating system, the GNU Fortran Compiler, GCC-2.95 (Win-32 version also known as G77; GNU Project, http://www.GNU.org/). Key pharmacokinetic parameters from the modeling are described in Figure 1.

The first-order conditional estimation method with η-ε interaction (FOCEI) was employed for all model runs. Individual estimates of pharmacokinetic parameters were obtained using POSTHOC (an empirical Bayesian estimation method). The random effect models sufficiently described the error distributions. For this analysis all interindividual errors were described by exponential error models on selected parameters (Equation 1).
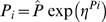
(1)where: *P_i_* is the true parameter value for individual *i*, 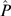 is the typical population value (geometric mean) of the parameter, *η^Pi^* are individual-specific interindividual random effects for individual *i* and parameter *P* and were assumed to be independently and identically distributed following a normal distribution with mean 0 and variance omega (ω) squared (*η∼N(0,* ω^2^ *)*).

The data could not support a full covariance block for the OMEGA matrix. Modeling began with the assumption of no covariance between interindividual random effects (diagonal ω matrix). Later, the covariance between clearance (CL) and volume of distribution in the central compartment (Vc) was estimated. For pharmacokinetic observations in this analysis, the residual error model was described by a combined additive and proportional error model (Equation 2).
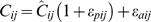
(2)where: *C_ij_* is the *j*th measured observation (plasma concentration) in individual *i*, 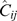 is the *j*th model predicted value (plasma concentration) in individual *i, ε_pij_* and *ε_aij_* are proportional and additive residual random errors, respectively, for individual *i* and measurement *j* and are assumed to be independently and identically normally distributed, following a normal distribution with mean 0 and variance sigma (σ) squared *(*ε*∼N(0,* σ*^2^)).* For each treatment arm, separate residual errors were explored. The pharmacokinetic models were evaluated for goodness of fit and were then subjected to predictive check model evaluation. For more detailed technical information on these methods, please see NONMEM user's guide [9].

After the structural pharmacokinetic model was established, known physiologic relationships were incorporated into the covariate-parameter models. For example, the change in physiologic parameters as a function of body size was both theoretically and empirically described by an allometric model (Equation 3) [10]

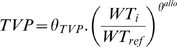
(3)where: the typical individual value of a model parameter (*TVP*) was described as a function of individual body weight (*WT_i_*), normalized by a reference weight (*WT_ref_*), which was 70 kg. *θ_TVP_* is an estimated parameter describing the typical pharmacokinetic parameter value for an individual with weight equal to the reference weight and *θ_allo_* is an allometric power parameter (which can be estimated or fixed to a value of 0.75 for clearances, and a value of 1 for anatomical volumes).

### Population Pharmacokinetic Model Development

Assessment of model adequacy and decisions about increasing model complexity were driven by the data and guided by goodness-of-fit criteria, including: (i) visual inspection of diagnostic scatter plots (observed vs. predicted concentration, residual/weighted residual vs. predicted concentration or time, and histograms of individual random effects; (ii) successful convergence of the minimization routine with at least 2 significant digits in parameter estimates; (iii) plausibility of parameter estimates; (iv) precision of parameter estimates; (v) correlation between model parameter estimation errors <0.95, and (vi) the Akaike Information Criterion (AIC), given the minimum objective function (OBJ) value and number of estimated parameters [9]. The criteria for successful runs were restricted to successful convergence using FOCE with interaction, good diagnostics for the model-fit for all data of the different treatment periods, and reasonable estimates for fixed and random effect parameters. Model evaluations included comparisons of the OBJ between hierarchical models. A decrease in OBJ corresponding to a chi-square distribution with α = 0.01 and degrees of freedom equal to the difference in the number of estimated parameters between the two models was used as the criterion for model comparisons.

Final model parameter estimates were reported with a measure of estimation uncertainty including the asymptotic standard errors (obtained from the NONMEM $COVARIANCE step). A limited covariate modeling approach emphasizing parameter estimation given the available data, rather than stepwise hypothesis testing, was implemented for this population pharmacokinetic analysis. The study population contained equal numbers of males and females. As such, age, weight and gender were explored as potential covariates. First, pre-defined covariate-parameter relationships were identified based on exploratory graphics, mechanistic plausibility of prior knowledge, and then a full model was constructed, with a fixed allometric relationship of body weight on clearance and volume parameters. Interindividual variability could not be incorporated on all fixed-effects parameters to get successful FOCE runs. For residual variance, a separate residual error was assigned for each of the treatment arms. A combined additive and proportional error model was used with 4 parameters to be estimated for the residual error. Various population models were evaluated, but only two models that best described the data (as determined by the log likelihood criterion and visual inspection) are presented. The first modeling approach was a population model that included all subjects. Because some of the modeling parameters and their variances were clearly not normally distributed, and showed asymmetric distribution, a mixture model was developed.

### Mixture Model

A second modeling approach was a population mixture model as it met our criteria for model adequacy and provided supporting evidence of the dichotomy of the observed individual data. Each subpopulation would have an associated submodel with different fixed or random effects. This model was adopted to accommodate the fact that only some of the individuals exhibited a pronounced increase in ivermectin bioavailability during the interaction arm of the study. It was preferred over a population model with and without outlier individuals, as it gave a better fit to the data as measured by change in OBJ, and met our criteria for a successful run in terms of a complete successful convergence with reasonable estimate for precision for both fixed and random effects.

### Model Evaluation

Model development was guided by various goodness-of-fit criteria, including diagnostic scatter plots. Checking of the individual fits was also employed as part of judging the model performance for each patient. The final model and parameter estimates were then investigated with the predictive check method. This method was similar to the previously described posterior predictive check, but assumes that parameter uncertainty is negligible, relative to interindividual and residual variance [11]. The basic premise is that a model and parameters derived from an observed data set should produce simulated data that are similar to the original observed data. The predictive check is a useful adjunct to typical diagnostic plots, in that the predictive check provides information about the performance of random-effects parameter estimates, whereas typical diagnostic plots are primarily informative about the fixed-effects parameter estimates. The predictive check model evaluation step was performed by using the final model and its parameter estimates to simulate data under the same experimental design of the original data.

One thousand Monte Carlo simulation replicates of the original data set were generated using the final non-mixture and mixture population pharmacokinetic models. Distributions of Cmax across all data simulations were compared with Cmax distribution in the observed data set. The simulated data from each of the 1000 virtual trials (18000 subjects for each treatment period) were assembled, and the similarity between the actual observed data and simulated data was examined by comparing the 95% predictions intervals of the simulated data with the original observed data.

## Results

**Figure 2:**
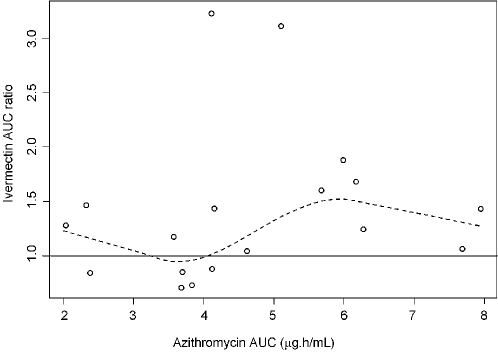
Ivermectin AUC ratio (AUC in interaction phase/AUC in baseline phase) versus azithromycin AUC in interaction phase. Solid line serves as a reference point of no change of ivermectin bioavailability; dotted line is Loess fit (local regression fit) to indicate lack of linear relationship. Circles are the observed individual values.

Assessment of the relationship between azithromycin and ivermectin by noncompartmental analysis showed that mean ivermectin AUC and Cmax was increased by 31% and 27%, respectively (see [5] for complete results). Visual inspection of the magnitude of ivermectin accumulation against azithromycin exposure in the interaction arm showed no obvious relationship (Figure 2), and a very low Pearson's r^2^ (0.03).

### Model

**Figure 3:**
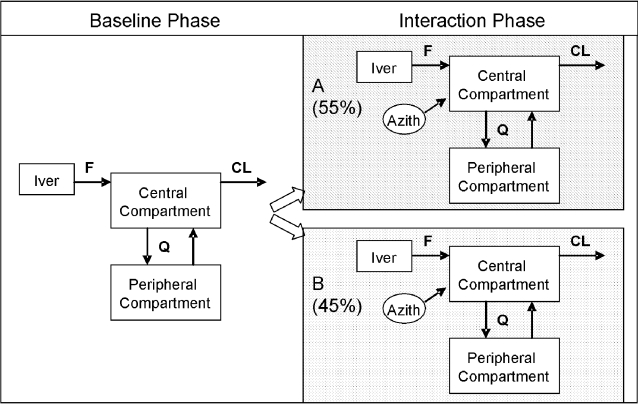
Two-compartment pharmacokinetic structural model for ivermectin. The best fit was obtained by models for two subpopulation (A and B), characterized by different F values, relative to the baseline model that included all subjects. Parameters: central and peripheral compartment volumes, total body clearance (CL), inter-compartmental clearance (Q), rate of absorption, and relative bioavailability (F). Note that albendazole was administered in both baseline and interaction phases.

Ivermectin concentration-time data were best described by a two-compartment pharmacokinetic model with first-order elimination and absorption (Figure 3). Covariance between CL and Vc elements of the OMEGA matrix was incorporated in the model. The use of different residual variance models stratified by the treatment with and without shared additive components was explored and incorporated into the structural model. Inclusion of age or gender as covariates did not contribute additional information for explaining pharmacokinetic variability based on OBJ differences in hierarchical models, model convergence, as well as diagnostic graphics. Therefore, none of these covariates was included as a covariate in the final population pharmacokinetic model. Importantly, the available data for this investigation contained a relatively small number of subjects and a limited age range, and so formal hypothesis (significance) testing for covariate effects was not considered.

**Figure 4:**
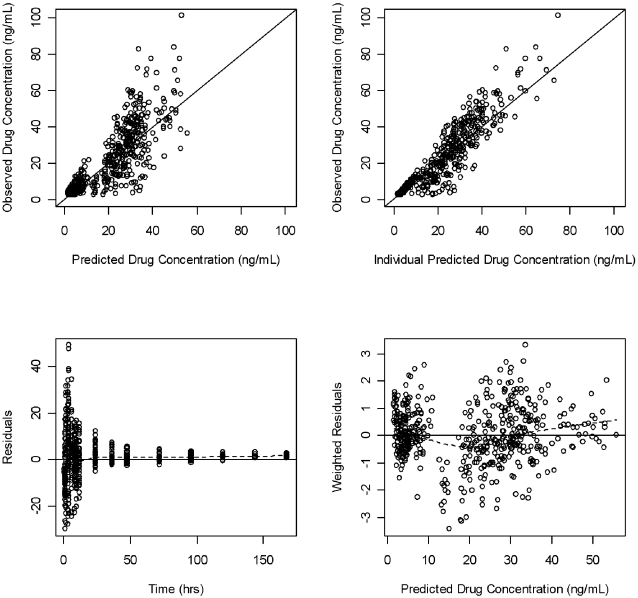
Goodness-of-fit plots for the final population mixture model. Observed versus predicted and individual predicted plasma ivermectin levels. The solid line represents the line of identity (top panels). Residual versus predicted plasma ivermectin levels and weighted residual versus time, (bottom panels).

The final non-mixture model had 7 fixed-effect parameters and 8 random-effect parameters as shown in Table 1. Population pharmacokinetic parameters (CL, Vc, Q, Vp; see Figure 1) were standardized to a 70 kg person using the allometric size model [10]. In parametric nonlinear mixed effects modeling, the distribution of *η*s is assumed to be normal (mean = 0, variance = *ω* ^2^). With each model developed, we checked the distribution of *η*s, and their mean values. The *η* distribution indicated a clear violation of the normality assumption. It was necessary to modify the original model to improve *η* distribution diagnostics. A mixture modeling approach was considered as the distribution of some of the pharmacokinetic parameters and inter-individual variabilities indicated a lack of homogeneity. The final mixture model had 9 fixed-effect parameters and 8 random-effect parameters as shown in Table 2. Goodness-of-fit plots for the final model are shown in Figure 4. The mixture model differed from the non-mixture model in only two parameters: one defining the difference between the two subpopulation in terms of bioavailability, the second defining the partition of the population between the two subpopulations. Using this approach, inter-individual variability distribution was modeled as two subpopulations (A and B). The unknown mixture distribution was estimated at an individual level. The estimate for each subpopulation included different fixed effects parameters, different variance parameters, estimation of fraction of individuals in each subpopulation, and each individual was assigned to the most likely subpopulation. The proportion of subjects in subpopulations A and B was estimated as 55% and 45%, respectively.

**Table 1:** Final Non-Mixture Model Parameter Estimates and Their Variabilities.

Parameter (unit)	Point Estimate	SEE	%RSE	%IIV
Fixed Effect Parameters				
θ_CL (L/h)_	11.8	3.87	32.79	
θ_Vc (L)_	195	123	63.07	
θ_Ka(1/h)_	0.24	0.11	45.83	
θ_Q (L/h)_	18.9	8.99	47.56	
θ_Vp (L)_	882	415	47.05	
θ _trt effect on Ka_	1.42	0.295	20.77	
θ _trt effect on F_	1.14	0.034	2.98	
Inter-individual Variability				
ω_CL_	0.023	0.109	473.913	15.165
Cov _CL, Vc_	−0.011	0.055	−500	10.488
ω_Vc_	0.063	0.093	147.619	25.099
ω_F1_	0.061	0.061	100	24.698
Residual Variability				
σ^2^_Baseline prop_	0.099	0.025	25.25	
σ^2^_Baseline add_	0.00			
σ^2^_co-admin prop_	0.081	0.033	40.74	
σ^2^_co-admin add_	0.00			

Point Estimate = Final Parameter Estimates for *θ*s, ω*s*, and σs; SEE = standard error of estimates; %RSE = relative standard error (100^*^(SEE/Estimate));

IIV(%CV) = interpatient variability (100^*^sqrt(Estimate for *ω^2^*)); *ω^2^*: random effect parameter that represents inter-patient variance; σ^2:^ random effect parameter that represents residual variance.

**Table 2:** Final Mixture Model Parameter Estimates and Their Variabilities.

Parameter (unit)	Point Estimate	SEE	%RSE	%IIV
Fixed Effect Parameters				
θ_CL (L/h)_	12.30	5.24	42.60	
θ_Vc (L)_	190	164	86.32	
θ_Ka(1/h)_	0.24	0.11	44.54	
θ_Q (L/h)_	19.0	8.61	45.32	
θ_Vp (L)_	841	412	48.99	
θ _trt effect on Ka_	1.38	0.16	11.67	
θ _F1 Subpop A_	0.99	0.24	23.84	
θ _F1 Subpop B_	1.37	0.16	11.90	
θ_mix proportions_	0.55	0.47	86.47	
Inter-individual Variability				
ω_CL_	0.04	0.19	497.31	19.29
Cov _CL, Vc_	0.01	0.08	1605.11	27.80
ω_Vc_	0.08	0.12	158.91	7.13
ω_F1_	0.07	0.09	127.76	26.57
Residual Variability				
σ^2^_Baseline prop_	0.09	0.02	25.28	
σ^2^_Baseline add_	0.00			
σ^2^_co-admin prop_	0.08	0.02	29.40	
σ^2^_co-admin add_	0.00			

See Table 1 for explanation of abbreviations.

### Population Pharmacokinetic Analysis

**Figure 5:**
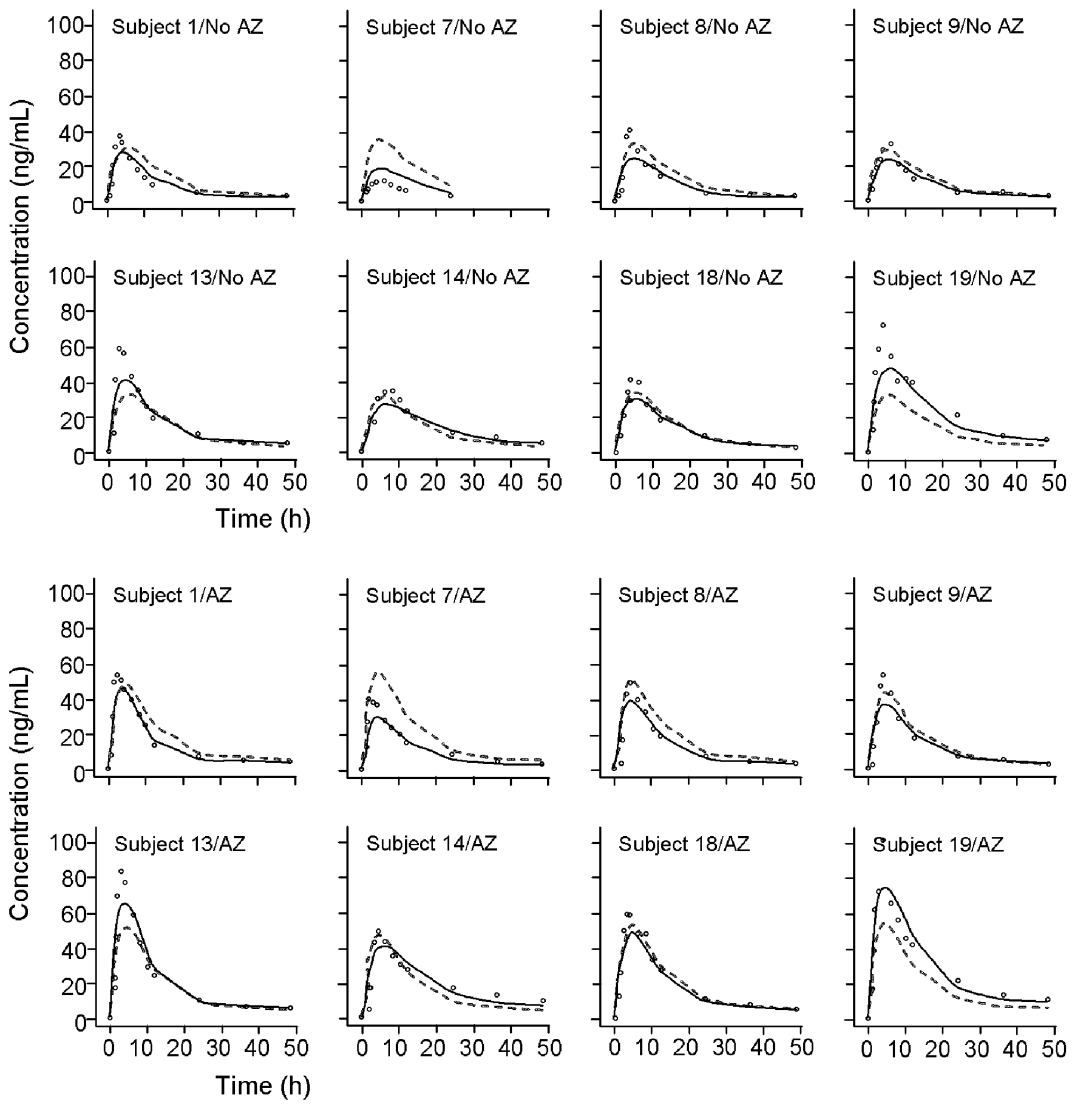
Observed, population predicted, and individual predicted ivermectin concentrations of individual subjects following ivermectin alone (No AZ) and after co-administration with azithromycin (AZ) for Subpopulation B, where increased bioavailability is observed in the interaction period. The solid line represents the fit predicted by the typical pharmacokinetic mixture model parameters. The dashed line shows the fit of the post hoc estimates of the population model. Circles represent the observed concentrations.

**Figure 6:**
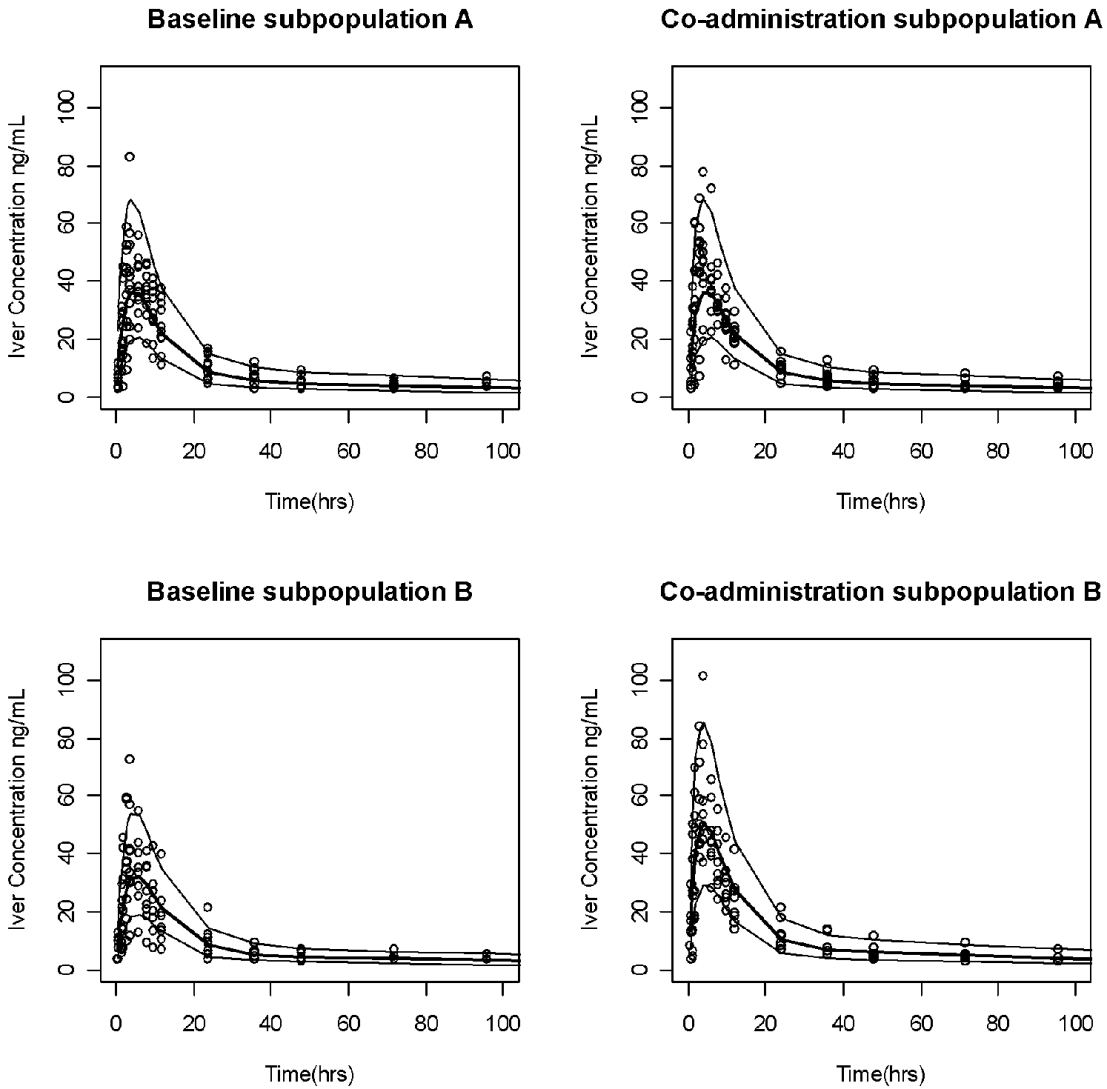
Observed data plotted as individual points. The solid center lines represent the median values of the 1000 simulated data sets, whereas the upper and lower lines represent the 97.5th and 2.5th quantiles of the simulated data, respectively.

Both the population and individual predictions adequately described the AUC profiles for each subject (Figure 5), as displayed by the baseline and interaction phases for subpopulation B. A similar fit of individual data was observed for Subpopulation A (data not shown). Figure 6 displays median, 97.5th, and 2.5th quantiles of the simulated data as lines with the observed data plotted as individual points. Less than 5% of the observed data were outside these 95% prediction intervals. No biased pattern or any tendency for over- or underestimation was noted for the different treatment periods, or for the two subpopulations. This finding gives confidence in the model performance in predicting the expected ivermectin exposures under different circumstances.

### Simulations

**Figure 7:**
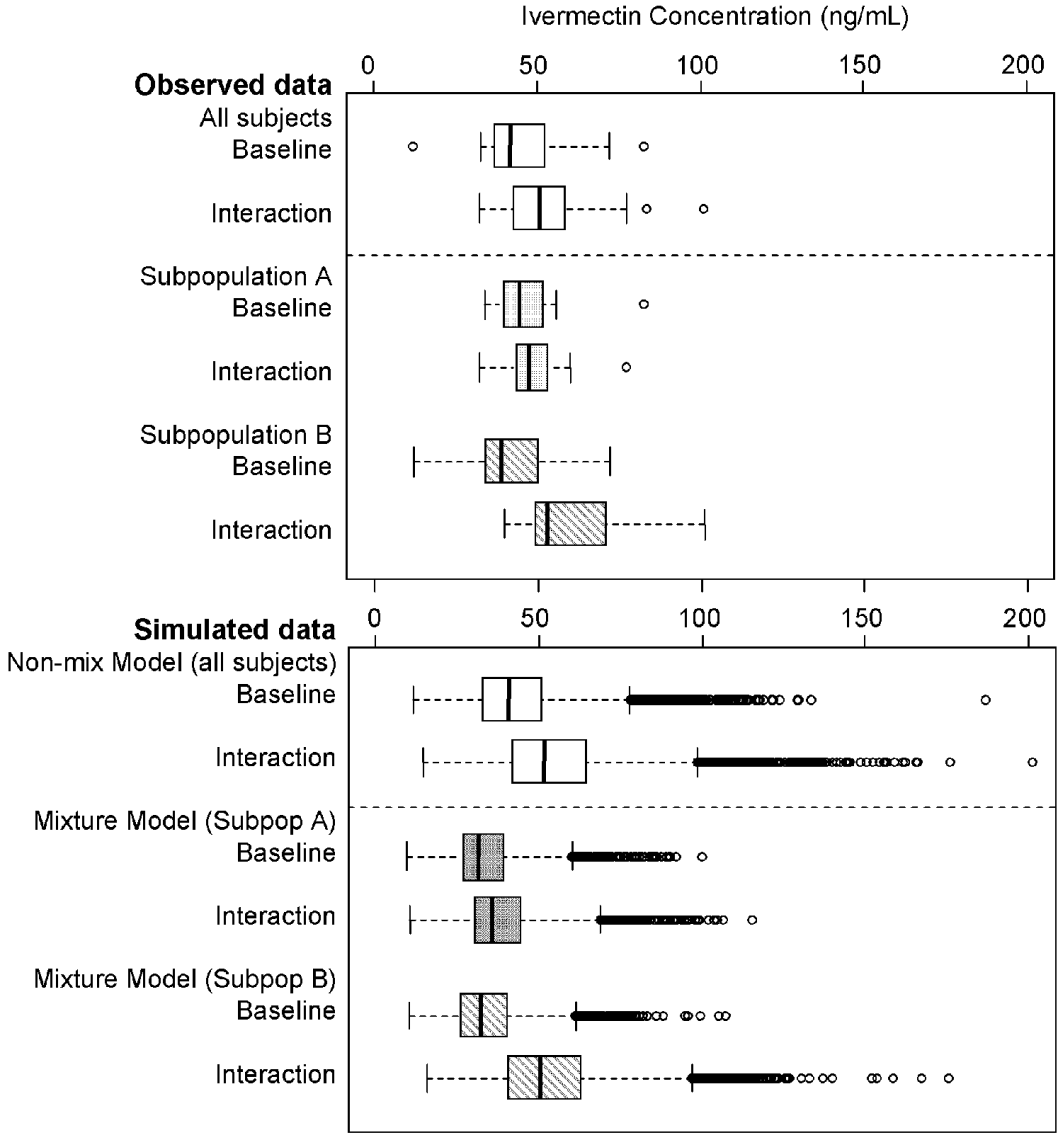
Upper panel: Observed maximum ivermectin concentration data in baseline and interaction arms from all subjects (open boxes) and from subpopulations A and B (shaded and hatched boxes). Lower panel: Maximum concentration data from 1000 simulation replicates using the non-mixture model in all subjects (open boxes) and from the mixture model in subpopulations A and B (shaded and hatched boxes). The line in the interior of the box denotes the median, the bottom and top edges denote the first and third quartiles, respectively. The lines from the top and bottom edges extend to 1.5 times the interquartile range. Values exceeding the interquartile range are plotted as individual points.

Simulated maximum concentrations for each individual's Cmax values were summarized across 1000 simulation replicates of the original population pharmacokinetic database and plotted as box plots (Figure 7). The upper panel shows box plots of the observed ivermectin Cmax for baseline and interaction periods for all subjects, and for the two subpopulations. The lower panel shows box plots for ivermectin Cmax from 1000 simulated trials for the non-mixture model (all subjects), and the mixture model (subpopulations A and B). The mixture model pattern predictions for the two subpopulations were very consistent with the observed data [5]. Extreme values were: non-mixture model: 201.2 ng/mL; mixture model subpopulation A: 115.3 ng/mL; B: 175.5 ng/mL.

## Discussion

There are a number of interesting findings from this analysis of data from an interaction study of ivermectin and azithromycin. This is the first published population model of ivermectin pharmacokinetics. It demonstrates the utility of population mixture modeling as an approach to explore drug interactions, especially where there may be population heterogeneity. The mechanism for the interaction was identified (an increase in bioavailability in one subpopulation). The model was used to simulate multiple clinical trials, to identify the maximum exposures that might be observed during co-administration, which permits comparison with previously published safety and pharmacokinetic data.

Ivermectin has been approved for use in humans for 2 decades, yet relatively limited pharmacokinetic data have been published. Recent studies using modern assay methods have characterized its pharmacokinetics using noncompartmental methods in the context of drug combination studies for treatment of onchocerciasis and lymphatic filariasis [12]–[14], or in high doses for treatment of head lice [7]. The calculated model parameters are in close agreement with those determined using noncompartmental methods [5]. A two compartment model is consistent with the disposition of ivermectin in man and other species, with a high volume of distribution into a peripheral compartment [15]. Ivermectin is metabolized extensively in the liver via cytochrome P450 isozyme (CYP) 3A4 [16]. It is both a substrate for the transporter P-glycoprotein (Pgp) [17],[18], as well as a moderately potent Pgp inhibitor at concentrations consistent with clinical exposures in the present study (IC_50_ 0.18–0.4 µM; [19],[20]).

The variability of the magnitude of change in ivermectin pharmacokinetics observed in the interaction phase [5] complicated the interpretation of the presence or absence of a drug interaction, as the response was very inconsistent among individuals. One of the objectives of this analysis was to explore how nonlinear mixed-effects modeling could be used to analyze such heterogeneous and highly variable experimental data from a relatively small number of subjects, with intensive pharmacokinetic sampling. The initial non-mixture model provided an adequate description of ivermectin pharmacokinetic data, however interindividual variability was not homogeneous and could not be explained by the available covariates. A mixture model was able to resolve this, and provided an explanation for the observed differences in bioavailability seen in the clinical study. Mixture modeling assumes two or more subpopulations exist, rather than a single homogeneous one [21], and the final model has two additional fixed parameters, one relating to subpopulation differences in ivermectin bioavailability, and the other defining the two subpopulations.

The final mixture model provided a good description of ivermectin data from both treatment periods. Goodness-of-fit criteria revealed that the final model was consistent with the observed data and that no systematic bias remained. The data points (Figure 4) are scattered closely and randomly around the line of identity, and the homogenous and random distributions of weighted residuals indicate the error model was suitable for describing the variance of the data. The model evaluation results provided evidence that both the fixed-effects and random-effects components of the final model were reflective of the observed data as well. The fact that less than 5% of the data were located outside the 2.5-97.5th quantile range suggests that the model accurately describes the central tendency and the variability of the data for the two subpopulations and for the two treatment periods, despite the large number of parameters and the low number of patients who participated in the study. The predictive check shows there is no bias at any phase of the pharmacokinetic profile, which makes the model useful in predicting ivermectin blood concentrations, when given alone or co-administered with azithromycin.

Typically, a mixture modeling approach would not be considered at the outset of a population pharmacokinetic analysis. Because of the unexplained remaining variability (see above), in the present analysis, the following decision rules were used in the evaluation of the mixture model: (i) The Estimation step and Covariance step terminated successfully; (ii) 95% CI for Mixture partition did not include 0 nor 1; and (iii) the change in the OBJ between mixture and non-mixture models was >5.99 (χ^2^; p<0.05, 2df). In the present analysis, the difference was 19.8.

The mixture model identified the interaction between azithromycin and ivermectin to be due to changes in bioavailability in Subpopulation B. Their mean estimate of bioavailability (F) was 1.37 relative to baseline, whereas F was unchanged for Subpopulation A (0.97). Inspection of noncompartmental data for Subpopulation B were consistent, showing higher Cmax and earlier Tmax values (Cmax A: 54.3 ng.h/mL; B: 67.8 ng.h/mL; Tmax A: 4.1 h; B: 3.4 h). There were no differences in apparent clearance or volume of distribution. However the mechanism for the increase in bioavailability is unclear. Azithromycin, like ivermectin, is a substrate for Pgp, however it has minimal inhibitory effects on this transporter in vitro [20]. Although ivermectin is extensively metabolized by CYP3A4 [16], azithromycin has no inhibitory activity against this enzyme [22]. There are no other plausible metabolic or transporter mechanisms that could explain an interaction, and no clinical covariates were identified that characterized either subpopulation. In addition, mean pharmacokinetic parameters of ivermectin were similar in both subpopulations in the baseline phase (mean AUC A: 1019; B: 805 ng.h/mL; Cmax A: 52; B: 45 ng/mL; Tmax A: 5.3; B: 4.8h).

The model was used to simulate the range of peak ivermectin concentrations that might be encountered if azithromycin and ivermectin were co-administered. These simulated data were then compared with the Cmax data reported in the high-dose ivermectin safety study [7]. The median simulated Cmax data (46.0, 34.1 and 40.3 ng/mL for non-mixture model, mixture models A and B respectively) were approximately 5–7-fold lower than the 261 ng/mL value reported by Guzzo et al [7]. Indeed, the most extreme individual simulated values (201.2, 115.3 and 175.5 ng/mL for non-mixture model, mixture models A and B respectively) were still lower than the mean value reported in the high-dose study [7]. These data give a high level of confidence that peak exposures that are predicted to occur if ivermectin and azithromycin were co-administered would never exceed mean values seen under high dose conditions [7], and which in this study were safe and well tolerated. In the Amsden et al interaction study [5], ivermectin was dosed with food (a high-fat breakfast). Food has been shown to increase the bioavailability of ivermectin over 2-fold [7]. Because dosing of patients in Africa is unlikely to be with high fat meals, extreme peak ivermectin concentrations would be half of those reported in the simulation.

Interestingly, simulations from both the mixture model and the non-mixture model had generally similar predictions of ivermectin exposures (average estimates and variability). Both models confirmed that the maximum concentration achieved in the interaction phase would not exceed 201 ng/mL (Figure 7). In spite of adding two parameters to the non-mixture model; the final parameter estimates for both models were very similar (Tables 1 and 2). The inflation of variability and projections of extreme values for both sets of simulations is a consequence of using 1000 replicates, where the chances of sampling from the very extreme values of random error distributions are more probable. However predicting extreme high values, even if they are very rare, is very useful from a safety perspective, and provide a “worst case” scenario of any extreme high exposures that might be encountered in a clinical setting/trial during co-administration.

There are several important caveats to this analysis. The data collected from the drug interaction study was not intended for population analysis, and a larger data set would have been desirable. The use of a mixture model could be criticized on the basis that random variations in the data could be ascribed *post hoc* to population differences. Indeed, although the mixture model identified two populations on the basis of different effects on bioavailability, it is unclear mechanistically what this difference might be due to. Finally, modeling and simulation can advise but cannot supplant clinical data. The findings from this study should be confirmed in further clinical or pharmacokinetic studies.

In conclusion, this analysis demonstrates the utility of a population model approach to analyze drug interaction data. The mechanism for the interaction was identified (an increase in bioavailability in one subpopulation). The model was also used to simulate multiple clinical trials, to identify the maximum exposures that might be observed during co-administration, and provides confidence that the peak ivermectin exposures would never exceed mean exposures that have previously been shown to be safe and well tolerated.
